# The future of physician advocacy: a survey of U.S. medical students

**DOI:** 10.1186/s12909-021-02830-5

**Published:** 2021-07-24

**Authors:** Susan Chimonas, Maha Mamoor, Anna Kaltenboeck, Deborah Korenstein

**Affiliations:** 1 Center for Health Policy & Outcomes at Memorial Sloan Kettering Cancer Center, 485 Lexington Avenue, New York, NY 10017 USA; 2 Center for Health Policy & Outcomes and chief of the General Internal Medicine Service at Memorial Sloan Kettering Cancer Center, New York, NY USA

**Keywords:** Medical education, Professional development, Physician advocacy

## Abstract

**Background:**

Advocacy is a core component of medical professionalism. It is unclear how educators can best prepare trainees for this professional obligation. We sought to assess medical students’ attitudes toward advocacy, including activities and issues of interest, and to determine congruence with professional obligations.

**Methods:**

A cross-sectional, web-based survey probed U.S. medical students’ attitudes around 7 medical issues (e.g. nutrition/obesity, addiction) and 11 determinants of health (e.g. housing, transportation). Descriptive statistics, Kruskal-Wallis tests, and regression analysis investigated associations with demographic characteristics.

**Results:**

Of 240 students completing the survey, 53% were female; most were white (62%) or Asian (28%). Most agreed it is very important that physicians encourage medical organizations to advocate for public health (76%) and provide health-related expertise to the community (57%). More participants rated advocacy for medical issues as very important, compared to issues with indirect connections to health (*p* < 0.001). Generally, liberals and non-whites were likelier than others to value advocacy.

**Conclusions:**

Medical students reported strong interest in advocacy, particularly around health issues, consistent with professional standards. Many attitudes were associated with political affiliation and race. To optimize future physician advocacy, educators should provide opportunities for learning and engagement in issues of interest.

**Supplementary Information:**

The online version contains supplementary material available at 10.1186/s12909-021-02830-5.

## Background

In recent decades, physician advocacy, particularly regarding social determinants of health and just distribution of resources, has been embraced as a core component of professionalism [1–3]. Medical organizations and codes of conduct frequently emphasize the importance of physician civic engagement [4, 5]. The American Medical Association (AMA), for example, urges physicians to “advocate for social, economic, educational, and political changes that ameliorate suffering and contribute to human well-being.” [6] Similarly, the Physician’s Charter asserts that “the medical profession must promote justice in the health care system, including the fair distribution of health care resources.” [7] And physicians themselves almost unanimously agree that community participation, political involvement, and collective advocacy are important professional duties [8].

Yet few physicians actually engage in these tasks [1]. In a 2004 survey, only a quarter of U.S. physicians reported participating politically (apart from voting) on local health issues [8, 9]. Indeed, physicians take part in community and political activities less frequently than the general population and other professionals with similar levels of education and income [10, 11]. While reasons for physicians’ low levels of engagement likely vary, [1] there is clearly more work to do in equipping physicians to participate in and contribute to civic life.

Educating medical students about their professional responsibility to advocate for health-related issues is essential to promoting more robust physician civic engagement in the future. Yet relatively little is known about students’ awareness of or interest in these vital topics. We therefore set out to understand medical students’ attitudes around civic engagement -- including their interests and future plans around health policy, their sense of responsibilities around healthcare access and costs, their attitudes toward different forms of public engagement, as well as specific issues of interest -- and to determine congruence with professional obligations. We also hypothesized that students would express stronger interest in advocacy around issues directly related to health and medical care (e.g. nutrition, addiction, care access) but lesser support for engagement around indirect determinants of health (e.g. transportation, education, economic inequality).

## Methods

### Survey administration

We conducted a cross-sectional, web-based survey of U.S. medical students. Participants were recruited from Student Doctor Network (SDN), a non-profit, online forum for current and future healthcare students and professionals. The survey link was posted on SDN’s Facebook and Twitter pages and on the SDN website’s homepage, online forums for allopathic and osteopathic medical students, and blog; the blog post was also distributed to self-identified medical students who had previously opted to receive SDN emails. The survey was anonymous, but participants could opt to provide their email address to enter a lottery for 1 of 20 $100 gift cards.

The survey launched on August 13, 2019, and closed on October 15, 2019. Responses were collected and managed using Research Electronic Data Capture (REDCap), a secure, web-based software platform hosted at Memorial Sloan Kettering Cancer Center (MSK) [12, 13]. Our findings are reported according to the Checklist for Reporting Results of Internet E-Surveys [14] (Supplemental Digital Appendix 1). The study was reviewed by the MSK Institutional Review Board (IRB) and deemed exempt.

### Survey instrument

We adapted some survey items from prior studies [8, 9, 15–19] and developed additional, novel questions focusing on study objectives (Supplemental Digital Appendix 2). A response to each question was required in order to proceed to the next. The survey included demographic items and several measures assessing participants’ interest in following or becoming involved in healthcare policy. Two measures tested their views of physicians’ responsibilities to patients around healthcare access and costs (providing care regardless of patients’ ability to pay, being aware of the overall costs of care they provide) [9, 19]. Additional items gauged participants’ attitudes toward 3 forms of physician civic engagement: [8, 19, 20] Community participation (providing health-related expertise to local populations), individual political engagement (being politically involved around health issues at the local, state, or national level), and collective advocacy (encouraging medical organizations to advocate for public health).

The survey also assessed participants’ support for individual or collective advocacy by physicians around 18 public priorities, adapted from recent national surveys of the U.S. population [17, 18]. Seven issues directly related to health and healthcare: healthcare costs, healthcare coverage for the uninsured, Medicare/Medicaid/Social Security, drug addiction and treatment, abortion laws/reproductive issues, nutrition/obesity/food safety, and disability rights. Eleven additional issues had connections to or implications for health: education, [21, 22] housing/homelessness, [23, 24] transportation, [25, 26] immigration, [27, 28] LGBTQ (lesbian, gay, bisexual, transsexual, and queer/questioning) issues, [29, 30] racial issues, [31, 32] economic issues, [33, 34] environmental issues, [35, 36] human rights, [37, 38] crime/criminal justice, [39, 40] and military/national security issues [41, 42].

Response options included Likert scales for agreement (strongly agree, agree, disagree, strongly disagree) and importance (very important, somewhat important, not important). We piloted a preliminary version of the survey with 15 medical students and internal medicine residents at Weill Cornell Medical Center and made minor changes to the survey based on their feedback and responses.

### Analysis

We used descriptive statistics to summarize participants’ demographics and attitudes. We used census zones to determine geographic region of participants’ schools. We used the Kruskal-Wallis test to evaluate associations between demographic characteristics (including gender, race, year in school, political identification, and anticipated future field) and attitudes around healthcare policy and forms of civic engagement. We also created a composite civic-mindedness score for each participant by averaging the strength of their responses (using scores of 1 for “not important,” 2 for “somewhat important,” and 3 for “very important”) to all 18 public-priorities questions (overall score); we similarly generated composite scores for the 7 issues directly related to health and healthcare (medical score), as well as for the 11 issues addressing indirect or social determinants of health (social score). We used univariate tests and multiple linear regression to evaluate associations between demographic characteristics and overall, medical, and social scores. All analysis was performed in Stata 14.2 [43].

## Results

There were 815 visitors to the SDN postings linked to the survey. Three hundred sixty-one unique individuals accessed the survey link, and 356 completed the first page to determine eligibility (based on attendance at an accredited U.S. medical school). Of 277 eligible participants, 240 completed the survey (view rate 44%; participation rate 77%; completion rate 87%) [14]. (Supplemental Digital Appendix 1).

Participant characteristics are shown in Table 1. Eighty-seven percent of participants were enrolled in MD programs. Approximately two thirds were 25–34 years old and white, and about half were women. Participant gender and race were similar to characteristics of U.S. medical students overall [44]. All geographic regions and years of medical school were represented, with slight overrepresentation of first-year students.

**Table 1:** Participant Characteristics (*N* = 240)

		N (%)
**Year in Medical School**	1	80 (33)
	2	52 (22)
	3	57 (24)
	4+	51 (21)
**Age (years)**	18–24	82 (34)
	25–34	150 (62.5)
	35–44	7 (2.9)
	45+	1 (0.4)
**Gender**	Male	112 (47)
	Female	127 (53)
	Non-binary	1 (0.4)
**Race**^**1**^	American Indian or Alaska Native	3 (1.3)
	Asian	68 (28.3)
	Black or African American	17 (7.1)
	Native Hawaiian or Other Pacific Islander	1 (0.4)
	White	149 (62.1)
	Other	16 (6.7)
**Hispanic, Latino or Spanish**	Yes	22 (9)
	No	218 (91)
**Degree Program**	MD	197 (82)
	DO	31 (13)
	MD/PhD	3 (1.2)
	MD/MPH	4 (1.6)
	MD/MS	5 (2.1)
**Geographic Region of participant school**^**2**^	Midwest	28 (12)
	Northeast	127 (53)
	South	29 (12)
	West	54 (23)
	Puerto Rico and Caribbean	3 (0.1)
**Anticipated future field**	Primary care	48 (20)
	Non-primary care clinical	147 (61)
	Non-clinical	4 (1.6)
	Undecided	41 (17)

^1^ Participants were asked to select “all that apply”

### Interest and intentions around healthcare policy

Three in four participants in our study were members of at least one medical organization addressing healthcare policy issues (e.g. American Medical Association, American Medical Student Association, American Medical Women’s Association). Nearly allreported following healthcare policy in the news (Table 2). Eight-7 % somewhat or strongly disagreed that healthcare policy will have little or no effect on their care of patients, with liberals twice as likely as conservatives to hold these views (*p* < 0.001). Most also planned to become involved or take leadership in healthcare policy issues as physicians; liberals and those intending to enter primary care were 2 and 6 times likelier, respectively, than others to express strong interest in policy involvement (*p* < 0.05).

**Table 2:** Participants’ interest and engagement in healthcare policy (*N* = 240)

Please indicate your level of agreement with the following statements:	Strongly Agree N (%)	Somewhat Agree N (%)	Somewhat Disagree N (%)	Strongly Disagree N (%)
I follow healthcare policy in the news.	56 (23.3)	141 (58.8)	37 (15.4)	6 (2.5)
Healthcare policy will have little or no effect on how I care for my patients.	4 (1.7)	27 (11.3)	67 (27.9)	142 (59.2)
I plan to become involved in healthcare policy issues as a physician.	73 (30.4)	119 (49.6)	44 (18.3)	4 (1.7)
I plan to take leadership in healthcare policy issues as a physician.	46 (19.2)	110 (45.8)	64 (26.7)	20 (8.3)

### Responsibilities around healthcare costs and access

The survey probed participants’ beliefs about physicians’ responsibilities around healthcare access and costs (Table 3). Three quarters agreed it is very important for physicians to *know the overall cost of the care they provide*. A large majority believed that it was very important for physicians to *provide necessary care regardless of the patient’s ability to pay*, although liberals were more likely than independents and conservatives to hold this view (*p* < 0.001).

**Table 3:** Participants’ assessments of civic responsibilities (*N* = 240)

How important is it for physicians to:	Very Important N (%)	Somewhat Important N (%)	Not Important N (%)
Provide necessary care regardless of the patient’s ability to pay.	195 (81.3)	43 (17.9)	2 (0.8)
Know the overall cost of the care they provide.	179 (74.6)	52 (21.7)	9 (3.8)
Encourage medical organizations to advocate for the public’s health.	183 (76.3)	47 (19.6)	10 (4.2)
Provide health-related expertise to local community organizations (e.g., school boards, parent-teacher organizations, athletic teams, local media).	137 (57.1)	91 (37.9)	12 (5.0)
Be politically involved (other than voting) in health-related matters at the local, state, or national level.	109 (45.4)	114 (47.5)	17 (7.1)

### Public roles: collective, community, and individual

The survey assessed participants’ attitudes toward 3 forms of civic involvement by physicians: collective advocacy, community participation, and political engagement (Table 3). Three quarters said that it was very important for physicians to *encourage medical organizations to advocate for the public’s health*. This measure correlated with political identification, with liberals more likely than independents and conservatives to hold this attitude (*p* < 0.001). More than half reported that it is very important for physicians to *provide health-related expertise to local community organizations*. First- and second-year students and those intending to enter primary care were more likely than others to express this opinion (*p* < 0.05). Fewer than half agreed that it is very important for physicians to be *politically involved in health-related matters at the local, state or national level*. Liberals were more likely than independents and conservatives to hold this view (*p* < 0.001).

### Issues of interest

The survey explored participants’ attitudes toward 18 public priorities (Table 4). Nearly all believed it was very important for physicians to individually or collectively advocate around drug addiction and treatment, healthcare coverage for the uninsured, and nutrition, obesity, and food safety. Large majorities also strongly favored professional engagement around healthcare costs, abortion laws and reproductive issues, human rights, disability rights, Medicare, Medicaid, and Social Security, and education. Similarly, most rated physician advocacy on racial issues, housing and homelessness, and LGBTQ issues as very important. Lesser support was evident for physician engagement around environmental issues, immigration, economic issues, crime and criminal justice, transportation, and military and national security issues .

**Table 4:** Issue priorities (*N* = 240)

Outside provision of direct patient care, how important is it that physicians, individually or collectively, advocate for:	Very Important N(%)	Somewhat Important N(%)	Not Important N(%)	Mean	Standard deviation	Variance
**Medical Issues**				**2.74**	**.3744**	**.1401**
Drug addiction and treatment	200 (83.3)	36 (15.0)	4 (1.7)	2.82	.4287	.1838
Healthcare coverage for the uninsured	195 (81.3)	39 (16.3)	6 (2.5)	2.79	.4672	.2183
Nutrition, obesity, and food safety	194 (80.8)	42 (17.5)	4 (1.7)	2.79	.4462	.1991
Healthcare costs	186 (77.5)	49 (20.4)	5 (2.1)	2.65	.4775	.2280
Abortion laws and reproductive issues	181 (75.4)	49 (20.4)	10 (4.2)	2.71	.5379	.2894
Disability rights	166 (69.2)	62 (25.8)	12 (5.0)	2.64	.5756	.3313
Medicare, Medicaid, Social Security	165 (68.8)	66 (27.5)	9 (3.8)	2.65	.5511	.3038
**Social Issues**				**2.35**	**.5350**	**.2863**
Human rights	167 (69.6)	59 (24.6)	14 (5.8)	2.64	.5909	.3492
Education	163 (67.9)	68 (28.3)	9 (3.8)	2.64	.5534	.3062
Racial issues	149 (62.1)	64 (26.7)	27 (11.3)	2.51	.6906	.4769
Housing and homelessness	138 (57.5)	81 (33.8)	21 (8.8)	2.49	.6532	.4266
LGBTQ issues	132 (55.0)	75 (31.3)	33 (13.8)	2.41	.7208	.5195
Environmental issues	113 (47.1)	96 (40.0)	31 (12.9)	2.34	.6966	.4853
Immigration	103 (42.9)	93 (38.8)	44 (18.3)	2.25	.7446	.5544
Economic issues	102 (42.5)	100 (41.7)	38 (15.8)	2.27	.7172	.5144
Crime and criminal justice	95 (39.6)	104 (43.3)	41 (17.1)	2.23	.7199	.5182
Transportation	86 (35.8)	117 (48.8)	37 (15.4)	2.20	.6876	.4728
Military and national security issues	52 (21.7)	115 (47.9)	73 (30.4)	1.91	.7179	.5153
**Overall**				**2.50**	**.4444**	**.1975**

*Overall civic-mindedness scores*, averaging the strength of participants’ responses (using scores of 1 for “not important,” 2 for “somewhat important,” and 3 for “very important”) to all 18 issues, had a mean of 2.5 and median of 2.6 (IQR: 2.28–2.83). *Medical scores*, based on participants’ assessments of the 7 issues directly related to health and healthcare (healthcare costs, healthcare coverage for the uninsured, Medicare/Medicaid/Social Security, drug addiction and treatment, abortion laws/reproductive issues, nutrition/ obesity/food safety, and disability rights), had a mean of 2.7 and a median of 2.9 (IQR: 2.57–3.00). *Social scores*, based on participants’ responses to the 11 issues with indirect connections to or implications for health (education, housing/ homelessness, transportation, immigration, LGBTQ issues, racial issues, economic issues, environmental issues, human rights, crime/criminal justice, and military/national security issues), had a mean of 2.4 and a median of 2.5 (IQR: 2.00–2.82). (Table 4).

Regression analysis indicated that liberal participants had higher overall, medical, and social scores than conservatives (*p* < 0.01). Nonwhite participants had higher medical scores than whites (*p* < 0.05); they also had higher overall and social scores, although this trend was not statistically significant. Kruskal-Wallis tests showed women and those intending to enter primary care to have higher overall and social scores than other participants (p < 0.05). However, our regression analysis did not find gender or intended future field to be a significant predictor, suggesting that political identification was driving these differences and capturing the variance in our model. (Table 5, Supplemental Digital Appendix 3).

**Table 5:** Univariate associations between student characteristics and civic engagement

		Overall civic engagement				Medical civic engagement				Social civic engagement			
		Mean Score	Median Score	SD	*p*-value**	Mean Score	Median Score	SD	p-value**	Mean Score	Median Score	SD	p-value**
**Gender**	Female (*n* = 127)	2.57	2.67	.41	0.0417*	2.78	3.00	.37	0.0812	2.43	2.55	.47	0.0324*
	Male (*n* = 112)	2.42	2.5	.47		2.69	2.86	.38		2.26	2.36	.58	
**Race**	White (*n* = 140)	2.44	2.53	.49	0.0517	2.68	2.86	.42	0.0310*	2.29	2.36	.57	0.0505*
	Non-white (*n* = 100)	2.58	2.67	.37		2.81	3.00	.28		2.44	2.55	.48	
**Political identification**	Conservative (*n* = 23)	2.12	2.28	.48	0.0001*	2.37	2.43	.50	0.0001*	1.96	2.00	.54	0.0001*
	Independent (*n* = 47)	2.27	2.33	.46		2.56	2.57	.39		2.09	2.09	.56	
	Liberal (*n* = 170)	2.62	2.72	.38		2.83	3.00	.30		2.48	2.55	.47	
**Future field**	Primary care (*n* = 48)	2.58	2.72	.46	0.0175*	2.75	2.93	.46	0.544	2.47	2.64	.49	0.0164*
	Non-primary care (clinical and non-clinical) (*n* = 151)	2.44	2.50	.45		2.70	2.86	.37		2.28	2.36	.55	
	Undecided (*n* = 41)	2.63	2.72	.36		2.85	3.00	.23		2.49	2.55	.49	

* Significant *P*-values

** P-values calculated using the Kruskal-Wallis test

## Discussion

Our findings can assist medical schools in preparing the next generation of physicians to more actively engage in civic life. Nearly all participants in our study expressed nascent interest in healthcare policy and civic engagement. Anticipating its relevance to their future practice, most stayed abreast of healthcare policy news and anticipated some degree of policy involvement as physicians. These results suggest that many students would welcome greater opportunities in medical school to learn about and participate in health policy issues.

Participant views of specific civic roles were more variable. Large majorities expressed a strong sense of professional obligation around the *just provision of clinical care*, from being cost-aware to treating patients who are unable to pay. These attitudes are similar to those of U.S. physicians. Notably, despite efforts to develop novel curricula, [45–47] these fundamental public health issues remain underemphasized in medical school environments. Our findings should encourage further efforts, as they suggest that many students might appreciate instruction around the challenges of caring for patients in a system where costs are frequently high and nontransparent and many patients are un- or under-insured.

About half of participants saw *civic engagement by individual physicians*, whether through community service or political involvement, as crucial. While similar to the views of practicing physicians in the U.S. [8], these attitudes stand in contrast to broad recognition of the important role of individual physicians in driving health system changes that will better meet the health needs of society [48]. More transparent role modeling of civic engagement by educators and better student access to meaningful advocacy opportunities could lead to greater student awareness of the importance of individual engagement and greater commitment to future action [49].

Most participants strongly supported *public health advocacy by medical organizations*. This finding mirrors the attitudes of U.S. physicians [8] and indicates that medical students would likely be interested in learning about how medical organizations have advocated around public health issues, as well as the obstacles (internal and external) to success. This would give students crucial insight into organized medicine’s mixed record in challenging or perpetuating the inequalities that pervade the U.S. healthcare system. It could also give students a greater appreciation for the enormous value of and need for civic engagement by individual physicians, who are often at greater liberty than medical organizations to critique inequities and participate in community and political affairs. To this end, schools should also highlight the numerous training and career opportunities available to medical professionals interested in advocacy.

Attitudes varied importantly by issue. Participants showed broad support for individual or collective physician advocacy around most of the public priorities included in our survey. Our findings regarding specific issues were consistent with results from physician surveys, [8, 9] with the exception of healthcare coverage for the uninsured, which was rated as very important by 81% of students in our study compared with 58% of practicing physicians in a 2006 study [8]. The overall similarity in attitudes is notable, given differences in generational attitudes, and may reflect a commonality of concerns among individuals with an interest in healthcare [50]. The greater emphasis on problems related to the uninsured in our study may related to increased public awareness of the issue [51].

As hypothesized, directly medical issues rated higher than those with indirect connections to or implications for health. This finding, while intuitive and broadly consistent with results from physician surveys, [8, 9] suggests that medical students in our study may not entirely understand the ways in which social determinants such as immigration, [27, 28] economic issues, [33, 34] transportation [25, 26] and crime/criminal justice issues [39, 40] can profoundly shape patients’ health. More, seemingly disparate issues are inevitably connected in that government budgets are finite, so choices in one area frequently have implications for others. For example, higher military expenditures are consistently associated with lesser funding for health and welfare programs [41, 42]. Medical schools should ensure that students fully grasp these and other ways in which non-medical issues systematically influence health.

Attitudes were generally stable across all years of medical school, but, as in physician surveys, [8, 9] many correlated to gender, race, future field, and political identification. Political identification was the most frequent and strongest predictor. Notably, conservatives in our study were more likely than liberal participants to doubt that healthcare policy would affect their care of patients, and conservatives had lower overall, medical, and social scores than liberals. These findings highlight the importance of ensuring that medical school curricula related to civic engagement recognize political diversity and remain attentive to appealing to concerns of students across the political spectrum. They also raise the question of whether some opportunities for education about and participation in civic engagement issues should be elective, providing opportunities for students to explore issues that best align with their interests.

Our findings with regard to race are also noteworthy and corroborate findings from physician surveys [8, 9]. Our sample was approximately one-third non-white; non-white participants identified predominantly as Asian (28%), with few (7%) identifying as Black or African American. Non-white participants had higher medical scores than whites, with a trend toward higher social and overall scores, as well -- suggesting consistently higher levels of civic engagement and perhaps broader interests compared to white students. The drivers of these attitudinal differences require further research, but may reflect the influence of diverse backgrounds and experiences.

However, as among medical students overall, Black students were underrepresented among our participants compared to the U.S. population [52]. Stronger representation of the views of underrepresented minority students is important for identifying issues for physician civic engagement that reflect the priorities of the broader population. More representative student populations might also engender greater student interest in a broader range of civic engagement issues, and ultimately a more engaged physician workforce. To optimize physician engagement, medical schools should redouble their efforts to recruit medical students who represent the diversity of the overall population.

This study has several limitations. Our modest sample size and online recruitment strategy raise the possibility that our findings may not be representative of all U.S. medical students. However, our study did capture attitudes from medical students from diverse geographical locations and with demographic characteristics similar to U.S. medical students overall [44]. In addition, we intentionally framed questions about specific issues in a neutral, apolitical way. However, some participants may still have perceived political bias in some items, such as “environmental issues” and “abortion laws and reproductive issues”; this perception may have influenced responses, particularly among conservative students. Finally, our survey was conducted prior to the COVID-19 pandemic, which has highlighted health disparities in minority populations, [53, 54] and the widespread Black Lives Matter protests following the murder of George Floyd [55]. These events may have influenced medical student attitudes toward civic engagement, particularly around issues with indirect links to health. Our findings do not reflect these potential shifts; future studies should evaluate the influence of these events on the attitudes of students and physicians.

## Conclusion

Medical students in our study reported keen interest in civic engagement and advocacy, particularly around issues directly involving health or healthcare services, which is generally consistent with professional standards. Many attitudes and interests are associated with political affiliation, race, gender, and intended future field. To optimize future physician advocacy, educators should provide opportunities for student learning and engagement in these vital matters.

### Practice points

Medical students want to learn about *just provision of care* and *advocacy by organizations*.

*Advocacy by individual physicians* is under-appreciated by students.

Students may incompletely understand how *social determinants* shape health.

Advocacy curricula should appeal to students *across the political spectrum.*

To ensure robust future advocacy, schools should recruit a *diverse and representative* student population.

## Supplementary Information

**Additional file 1: Supplemental Digital Appendix 1.** Checklist for Reporting Results of Internet E-Surveys (CHERRIES).

**Additional file 2: Supplemental Digital Appendix 2.** Survey instrument.

**Additional file 3.**

## Data Availability

The datasets generated during and/or analyzed during the current study are available from the corresponding author on reasonable request. We are developing another manuscript and prefer not to openly post the data until this work is complete.

## Abbreviations

AMA: American Medical Association
IRB: Institutional Review Board
LGBTQ: Lesbian, gay, bisexual, transsexual, and queer/questioning
MSK: Memorial Sloan Kettering Cancer Center
REDCap: Research Electronic Data Capture
SDN: Student Doctor Network
