# Supplementary material for: The future of physician advocacy: a survey of U.S. medical students
Source: BMC Med Educ. 2021 Jul 24;21:399. doi: 10.1186/s12909-021-02830-5 (PMC8310411; doi:10.1186/s12909-021-02830-5)
Supplement: Supplementary file 1 — Additional file 1: Supplemental Digital Appendix 1. Checklist for Reporting Results of Internet E-Surveys (CHERRIES). [file 12909_2021_2830_MOESM1_ESM.docx]

Supplemental Digital Appendix 1: Checklist for Reporting Results of Internet E-Surveys (CHERRIES)

| **Item Category** | **Checklist Item** | **Explanation** |
| --- | --- | --- |
| Design | Describe survey design | We adapted some items from prior studies and developed new items. Items included demographic items, items addressing attitudes toward drug pricing including self-assessed knowledge, education around drug pricing, and factors influencing drug pricing, and items that tested knowledge of drug prices and their determinants (Appendix). Response options included likert scales related to agreement (strongly agree, agree, disagree, strongly disagree) and importance (very important, somewhat important, not important), and true-false questions. |
| IRB (Institutional Review Board) approval and informed consent process | IRB approval | The project was reviewed by the MSK IRB, which determined the study to be exempt from review. |
|  | Informed consent | Participation was voluntary. The MSK IRB approved a waiver of informed consent; clicking on the survey was deemed adequate evidence of consent to participate. The survey invitation began with a description of physicians in public life followed by text inviting them to participate in the survey.:  “SDN and Memorial Sloan Kettering Cancer Center are partnering to conduct research on physician engagement, and want to hear from you: What public issues move you? Is medical school preparing you to participate in public life? We are interested in YOUR views and experiences. Please click here to complete our brief survey -- be entered in a lottery to win one of 10 $200 Amazon gift cards!” |
|  | Data protection | All data was managed through Research Electronic Data Capture (REDCap), a data management software system. REDCap is maintained on MSK-owned servers that are kept in a locked server room with appropriate environmental modifications (e.g., special air conditioning), supported by an uninterrupted power supply, and backed up nightly. All connections to REDCap use encrypted (SSL-based) connections. We did not collect any PHI. Subjects had the option of providing email addresses if they wished to collect an incentive. |
| Development and pre-testing | Development and testing | We piloted a preliminary survey among Weill Cornell Medical College residents in March 2019, with 15 residents providing written responses and 30 participating in a follow-up discussion. Survey items were adjusted based on their feedback. |
| Recruitment process and description of sample having access to the questionnaire | Open survey versus closed survey | Open survey. |
|  | Contact mode | The survey was distributed through Student Doctor Network (SDN), a non-profit online platform that provides free advising resources, tools, and peer-support forums. SDN includes nearly 700,000 members, including many medical students. The survey was made available on the SDN Medical Student blog and emailed to SDN members who opted in to receiving emails. |
|  | Advertising the survey | The survey was announced on the SDN website on a banner that stated:  “What public issues move you? Complete a brief research survey to be entered in a lottery to win one of 20 $100 Amazon gift cards!” |
| Survey administration | Web/Email | Survey posted on a website. SDN members were also emailed with a link to the survey invitation on the SDN website. Responses were automatically captured on REDCap. |
|  | Context | SDN comprises of nearly 700,000 members and the community includes most doctoral-level health fields DDS, DMD, MD, DO, OD, PharmD, DPM, PsyD, PhD, AuD, OTD, DPT, PhD, and DVM. |
|  | Mandatory/voluntary | Voluntary. |
|  | Incentives | Chance to win one of 20 $100 Amazon gift cards. |
|  | Time/Date | The survey was conducted between August 13, 2019 and October 15, 2019. |
|  | Randomization of items or questionnaires | None. |
|  | Adaptive questioning | The lead-in form to the survey was two CAPTCHA questions to ensure the user completing the survey is human. The first question of the survey itself asked about year in medical school. If the participant chose “I am not currently a medical student” the survey terminated. |
|  | Number of items | The survey is included as an Appendix and was comprised of 37 questions. |
|  | Number of screens (pages) | 10 pages including the lead in form and the thank you for completing the survey page. |
|  | Completeness check | The survey required response to each question in order to move on to the next page. |
|  | Review step | Respondents were unable to review and change their answers through a back button or a review step but could scroll up and down on each page. |
| Response rates | Unique site visitor | 815 unique site visitors (Number of blog visits). |
|  | View rate (Ratio unique survey visitors / unique site visitors) | 44% (361 clicked on the Captcha page/815 unique site visitors) |
|  | Participation rate (Ratio unique visitors who agreed to participate / unique first survey page visitors) | 77%  (277 completed the first page of the survey/361 clicked on the captcha page) |
|  | Completion rate (Ratio of users who finished the survey / users who agreed to participate) | 87%  (240 completed the survey/277 completed the first page of the survey) |
| Preventing multiple entries from the same individual | Cookies used | This feature was not used in the survey. |
|  | IP check | This feature was not used in the survey. |
|  | Log file analysis | This feature was not used in the survey. |
|  | Registration | NA. |
| Analysis | Handling of incomplete questionnaires | Only completed questionnaires were analyzed. |
|  | Questionnaires submitted with an atypical timestamp | NA |
|  | Statistical correction | NA |
