# Supplementary material for: The future of physician advocacy: a survey of U.S. medical students
Source: BMC Med Educ. 2021 Jul 24;21:399. doi: 10.1186/s12909-021-02830-5 (PMC8310411; doi:10.1186/s12909-021-02830-5)
Supplement: Supplementary file 2 — Additional file 2: Supplemental Digital Appendix 2. Survey instrument. [file 12909_2021_2830_MOESM2_ESM.pdf]

Supplemental Digital Appendix 2: Survey instrument

Physicians in public life

Please complete the survey below.

Thank you!

Please note that this survey requires a response to every question.

Year in medical school	◯ 1<br>◯ 2<br>◯ 3<br>◯ 4+<br>◯ I am not currently a medical student
What is your age?	◯ 18-24 years old<br>◯ 25-34 years old<br>◯ 35-44 years old<br>◯ 45+
Gender	◯ Female<br>◯ Male<br>◯ Non-binary
Which best describes your race? Select all that apply:	☐ American Indian or Alaska Native<br>☐ Asian<br>☐ Black or African American<br>☐ Native Hawaiian or Other Pacific Islander<br>☐ White<br>☐ Other
Are you of Hispanic, Latino, or Spanish origin?	◯ Yes<br>◯ No
Which medical school do you attend?	__________________________________
Current degree program	◯ MD<br>◯ DO<br>◯ MD/PhD<br>◯ MD/MPH<br>◯ MD/MS<br>◯ MD/JD
Anticipated med school debt	◯ 0<br>◯ $1-99k<br>◯ $100-199k<br>◯ $200-299k<br>◯ $300-399k<br>◯ $400k+
Anticipated future field	◯ Primary care<br>◯ Non-primary care clinical<br>◯ Non-clinical<br>◯ Undecided

Overall, how would you characterize yourself politically?	◯ Very conservative<br>◯ Somewhat conservative<br>◯ Independent/moderate<br>◯ Somewhat liberal/progressive<br>◯ Very liberal/progressive
Are you currently a member of any of these medical organizations? Please select all that apply:	☐ American Medical Association (AMA)<br>☐ American Medical Student Association (AMSA)<br>☐ American Medical Women's Association (AMWA)<br>☐ American Association of Medical Colleges (AAMC)<br>☐ Specialty society (e.g. ACP, ACS, AAFP)<br>☐ Other (please specify)<br>☐ None of the above
Other (please specify)	__________________________________

Please indicate your level of agreement with the following statements:

	Strongly Disagree	Somewhat Disagree	Somewhat Agree	Strongly Agree
I follow healthcare policy in the news.	◯	◯	◯	◯
I plan to become involved in healthcare policy issues as a physician (e.g., through donations, volunteering, etc).	◯	◯	◯	◯
I plan to take leadership in healthcare policy issues as a physician.	◯	◯	◯	◯
Healthcare policy will have little or no effect on how I care for my patients.	◯	◯	◯	◯

How important is it for physicians to:

	Very important	Somewhat important	Not important
Provide necessary care regardless of the patient's ability to pay.	○	○	○
Know the overall cost of the care they provide.	○	○	○
Provide health-related expertise to local community organizations (e.g., school boards, parent-teacher organizations, athletic teams, local media).	○	○	○
Be politically involved (other than voting) in health-related matters at the local, state, or national level.	○	○	○
Encourage medical organizations to advocate for the public's health.	○	○	○

Outside provision of direct patient care, how important is it that physicians, individually or collectively, advocate for the following:

	Very important	Somewhat important	Not important
Healthcare costs	○	○	○
Healthcare coverage for the uninsured	○	○	○
Medicare, Medicaid, and Social Security	○	○	○
Drug addiction and treatment	○	○	○
Abortion laws and reproductive issues	○	○	○
Nutrition, obesity, and food safety	○	○	○
Education	○	○	○
Housing and homelessness	○	○	○
Transportation	○	○	○
Immigration	○	○	○
LGBTQ issues	○	○	○
Racial issues	○	○	○
Disability rights	○	○	○
Economic issues	○	○	○
Environmental issues	○	○	○
Human rights	○	○	○
Crime and criminal justice	○	○	○
Military and national security issues	○	○	○

How would you rate your UNDERSTANDING of the factors that influence drug pricing?

◯ Excellent ◯ Good ◯ Fair ◯ Poor

How would you rate the QUANTITY of instruction at your medical school about factors that influence drug pricing?

◯ Too much ◯ Adequate ◯ Too little ◯ I have not received any such instruction

How would you rate the QUALITY of instruction at your medical school about factors that influence drug pricing?

◯ Excellent ◯ Good ◯ Fair ◯ Poor ◯ I have not received any such instruction

How important is it for physicians to understand the factors that influence drug pricing?

◯ Very important ◯ Somewhat important ◯ Not important

What proportion of U.S. prescriptions are for brand-name drugs?

◯ < 20% ◯ 20-39% ◯ 40-69% ◯ 70-90% ◯ >90% ◯ Don't know

What proportion of U.S. drug spending is for brand-name products?

◯ < 20% ◯ 20-39% ◯ 40-69% ◯ 70-90% ◯ >90% ◯ Don't know

Identify whether each statement below is TRUE or FALSE.

	True	False	Don't know
Drug prices in the U.S. are about the same as prices in other developed countries.	○	○	○
Drug PRICES in the U.S. are rising faster than inflation.	○	○	○
U.S. drug SPENDING is rising less than other health care spending.	○	○	○
Drug prices are NOT correlated with effectiveness.	○	○	○
Drug prices are correlated with research and development costs.	○	○	○
The rise in U.S. drug SPENDING is driven more by increased utilization than by higher prices.	○	○	○
Drugs with higher retail prices always cost health plans more than drugs with lower retail prices.	○	○	○
When administering intravenous drugs to patients in clinics, physicians receive higher payments from insurers if they use lower-priced, more cost-effective drugs.	○	○	○

Choose the five GROUPS that MOST INFLUENCE a new drug's price. Indicate whether the remaining GROUPS are somewhat or not influential.

	Most influential (choose up to 5)	Somewhat influential	Not influential	Don't know
Drug companies	○	○	○	○
Pharmacies	○	○	○	○
Hospitals and clinics	○	○	○	○
Private practices	○	○	○	○
Professional medical societies	○	○	○	○
International pricing committees	○	○	○	○
Insurance companies	○	○	○	○
Pharmacy benefit managers	○	○	○	○
Wholesalers	○	○	○	○
Patient advocacy groups	○	○	○	○
Food and Drug Administration (FDA)	○	○	○	○
National Institutes of Health (NIH)	○	○	○	○
Medicare/Medicaid	○	○	○	○
Congress	○	○	○	○
Other-please specify	○	○	○	○

Other - please specify

____

Choose the five ISSUES that MOST INFLUENCE a new drug's price. Indicate whether the remaining ISSUES are somewhat or not influential.

	Most influential (choose up to 5)	Somewhat influential	Not influential	Don't know
Cost of the drug's development	○	○	○	○
Cost of marketing to doctors	○	○	○	○
Cost of marketing to consumers	○	○	○	○
Insurance company policies	○	○	○	○
Middleman in the drug distribution system	○	○	○	○
Financial incentives of healthcare providers	○	○	○	○
Government regulations	○	○	○	○
Consumer demand	○	○	○	○
Rarity of the disease/condition	○	○	○	○
Population burden of the disease/condition	○	○	○	○
Novelty of the drug's mechanism	○	○	○	○
Number of other drugs for the disease/condition	○	○	○	○
Safety and effectiveness of the drug	○	○	○	○
Safety and effectiveness of other drugs for the disease/condition	○	○	○	○
Other - please specify	○	○	○	○

Other - please specify

____

What would you like to learn more about? Please select all that apply:

- ☐ How drug prices are determined and by whom
- ☐ Factors and conditions that promote higher drug prices
- ☐ The consequences of rising drug prices for patients and/or society
- ☐ Potential solutions to higher drug prices
- ☐ Not interested in learning about drug pricing
- ☐ Other health policy issues (please specify)

Health policy issues you would like to learn more about

____

Please feel free to share any additional thoughts or comments about physician engagement in public issues.

____

If you would like to be entered into a lottery to win a $100 Amazon gift card, please provide your MEDICAL school email address. __________________________________

Thank you for completing the survey! Please click the submit button to submit your answers.

Thank you for your interest, but this survey is for current medical students only.
