# Supplementary material for: The future of physician advocacy: a survey of U.S. medical students
Source: BMC Med Educ. 2021 Jul 24;21:399. doi: 10.1186/s12909-021-02830-5 (PMC8310411; doi:10.1186/s12909-021-02830-5)
Supplement: Supplementary file 3 — Additional file 3. [file 12909_2021_2830_MOESM3_ESM.docx]

| **Supplemental Digital Appendix 3**  **Regression results for average MEDICAL score**  Observations: 240 | | | | | | |
| --- | --- | --- | --- | --- | --- | --- |
| Variable | Coefficient | Std. Error | t-Statistic | P>\|t\| | 95% Conf. Interval | |
| Female | -.0170634 | .0458814 | -0.37 | 0.710 | -.1074608 | .0733341 |
| Male | -.0944942 | .3428978 | -0.28 | 0.783 | -.7700858 | .5810974 |
| Non-white | .0956829 | .0453869 | 2.11 | 0.036 | .0062597 | .1851062 |
| Independent/moderate | .1713614 | .0868281 | 1.97 | 0.050 | .000289 | .3424338 |
| Very/somewhat liberal/progressive | .4452303 | .0778744 | 5.72 | 0.000 | .2917988 | .5986617 |
| Non primary care clinical and non-clinical | .0499044 | .0581659 | 0.86 | 0.392 | -.0646965 | .1645054 |
| Undecided | .1040244 | .0728318 | 1.43 | 0.155 | -.0394718 | .2475206 |
| Constant | 2.3067 | .0923089 | 24.99 | 0.000 | 2.124829 | 2.48857 |
| F(7,232) | 8.84 |  | | | | |
| Prob > F | 0.0000 |  |  |  |  |  |
| R-squared | 0.2105 |  |  |  |  |  |
| Adjusted R-squared | 0.1867 |  |  |  |  |  |
| Root MSE | .33762 |  |  |  |  |  |

A multiple linear regression was run to predict average scores of the importance of medically-related civic engagement issues from gender, race, political identification and future field. The model suggests that race statistically significantly predicts the average score of medical students responding to medically related civic engagement issues. The model also suggests that political identification statistically significantly predicts the average score of medical students responding to medically related civic engagement issues. We are 99% confident that we can reject the null hypothesis. We conclude that the R^2 does not equal zero and our regression model has some explanatory value. 21.05% of the variation is explained by our model.

| **Regression results for average SOCIAL score**  Observations: 240 | | | | | | |
| --- | --- | --- | --- | --- | --- | --- |
| Variable | Coefficient | Std. Error | t-Statistic | P>\|t\| | 95% Conf. Interval | |
| Female | -.0673363 | .0681185 | -0.99 | 0.324 | -.2015493 | .0668767 |
| Male | .3808346 | .5050121 | 0.75 | 0.452 | -.614184 | 1.375853 |
| Non-white | .1111644 | .0669386 | 1.66 | 0.098 | -.0207238 | .2430526 |
| Independent/moderate | .0829498 | .1280085 | 0.65 | 0.518 | -.1692636 | .3351632 |
| Very/somewhat liberal/progressive | .4419657 | .1147916 | 3.85 | 0.000 | .2157935 | .66813 |
| Non primary care clinical and non-clinical | -.0684395 | .0857688 | -0.80 | 0.426 | -.2374287 | .1005497 |
| Undecided | -.1189549 | .2645395 | -0.45 | 0.653 | -.6401736 | .4022638 |
| Constant | 2.04808 | .1359524 | 15.06 | 0.000 | 1.780214 | 2.315945 |
| F(7,232) | 5.72 |  | | | | |
| Prob > F | 0.0000 |  |  |  |  |  |
| R-squared | 0.1653 |  |  |  |  |  |
| Adjusted R-squared | 0.1364 |  |  |  |  |  |
| Root MSE | .49723 |  |  |  |  |  |

A multiple linear regression was run to predict average scores of the importance of socially related civic engagement issues from gender, race, political identification and future field. The model suggests that political identification statistically significantly predicts the average score of medical students responding to socially related civic engagement issues. We are 99% confident that we can reject the null hypothesis. We conclude that the R^2 does not equal zero and our regression model has some explanatory value. 16.53% of the variation is explained by our model.

| **Regression results for average OVERALL score**  Observations: 240 | | | | | | |
| --- | --- | --- | --- | --- | --- | --- |
| Variable | Coefficient | Std. Error | t-Statistic | P>\|t\| | 95% Conf. Interval | |
| Female | -.050616 | .0554966 | -0.91 | 0.363 | -.1599458 | .0587427 |
| Male | .1953403 | .4114367 | 0.47 | 0.635 | -.6153079 | 1.005989 |
| Non-white | .1039822 | .0545353 | 1.91 | 0.058 | -.003468 | .2114324 |
| Independent/moderate | .1192259 | .1042893 | 1.14 | 0.254 | -.086254 | .3247058 |
| Very/somewhat liberal/progressive | .4448083 | .0935214 | 4.76 | 0.000 | .2605442 | .6290723 |
| Non primary care clinical and non-clinical | -.0237985 | .0698764 | -0.34 | 0.734 | -.1614751 | .1138782 |
| Undecided | .028594 | .2155221 | 0.13 | 0.895 | -.3960464 | .4532343 |
| Constant | 2.148361 | .1107613 | 19.40 | 0.000 | 1.93013 | 2.366593 |
| F(7,232) | 7.08 |  | | | | |
| Prob > F | 0.0000 |  |  |  |  |  |
| R-squared | 0.1969 |  |  |  |  |  |
| Adjusted R-squared | 0.1690 |  |  |  |  |  |
| Root MSE | .4051 |  |  |  |  |  |

A multiple linear regression was run to predict average scores of the importance of the overall related civic engagement issues from gender, race, political identification and future field. The model suggests that political identification statistically significantly predicts the average score of medical students responding to the overall related civic engagement issues. We are 99% confident that we can reject the null hypothesis. We conclude that the R^2 does not equal zero and our regression model has some explanatory value. 19.69% of the variation is explained by our model.
